# Autism Spectrum Disorder and Comorbid Eating Disorders: Case Presentation of a 14-Year-Old Male with Carotenemia

**DOI:** 10.1192/j.eurpsy.2025.1135

**Published:** 2025-08-26

**Authors:** J. González Benito, D. Durán López, C. Delgado Torres

**Affiliations:** 1 Psiquiatría, Servicio Canario de Salud, Santa Cruz de Tenerife, Spain

## Abstract

**Introduction:**

Atypical eating behaviors are more common in children and adolescents with Autism Spectrum Disorder (ASD) compared with neurotypical development. Cognitive inflexibility in ASD can result in rigid food-related rules and preoccupation with eating, similar to Eating Disorders (ED). Several studies have focused on cataloging these atypical eating behaviors, such as Zickgraf H. et al. (2019). [Figure 1].

**Objectives:**

Case presentation attended at the outpatient clinic of the Hospital Universitario Nuestra Señora de Candelaria (Tenerife).

Literature review on atypical eating behaviors in ASD patients and their comorbidity with EDs.

**Methods:**

A 14-year-old male, with no prior mental health history, showed normal social and academic adaptation but had a notable deficit in social smiling and persistent food texture rejection. He lives with his mother and has a close relationship with his paternal uncle, a personal trainer and role model.

He was referred to mental health services after multiple episodes of agitation, triggered by disruptions in his eating and exercise routines. Over the past year, he developed an intense focus on physical training practicing calisthenics several times a day, in addition to rejecting high-calorie foods, opting for vegetables (carrots and pumpkins). A conversation with soccer teammates, where comments about his body were made, may have acted as a trigger.

**Results:**

Physical Examination: Yellowish skin pigmentation, particularly on palms and nasolabial folds, not affecting sclera.BMI: 21.7.

Mental Status Examination: Aprosodic speech, limited eye contact. Marked rigidity with daily routines; severe anxiety when disrupted. Irritability related to eating and exercise. Food restriction focused on fruits and vegetables (carrots, pumpkins); interest in “being healthier” (muscle gain, fat loss).No purging or binge eating behaviors.

Psychometric Tests: ASSQ, ASAS, WISC-R suggest Autism Spectrum Disorder, Level 1, with average intellectual functioning.

Differential Diagnosis: Biochemical tests ruled out jaundice, liver disease, hypothyroidism, and diabetes. Diagnosis of carotenemia made by exclusion.

Diagnostic Interviews (EDI-III):Symptoms consistent with Avoidant/Restrictive Food Intake Disorder (ARFID).

**Image 1:**

**Figure:**
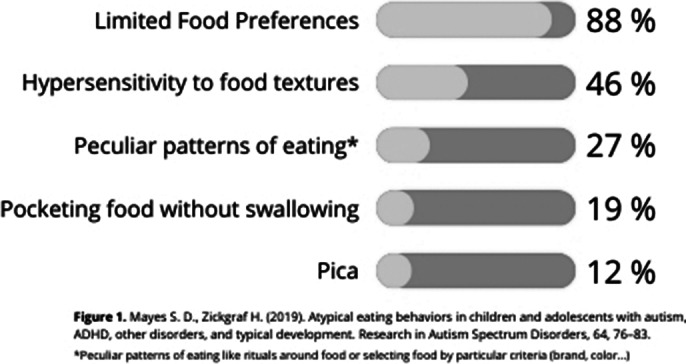

**Image 2:**

**Figure:**
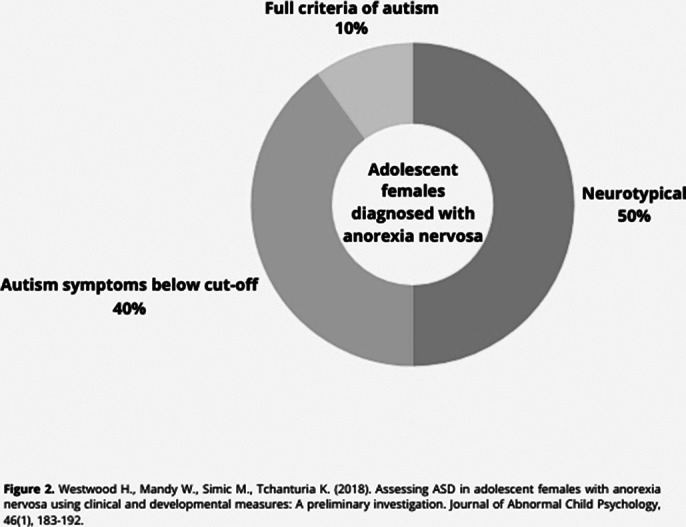

**Image 3:**

**Figure:**
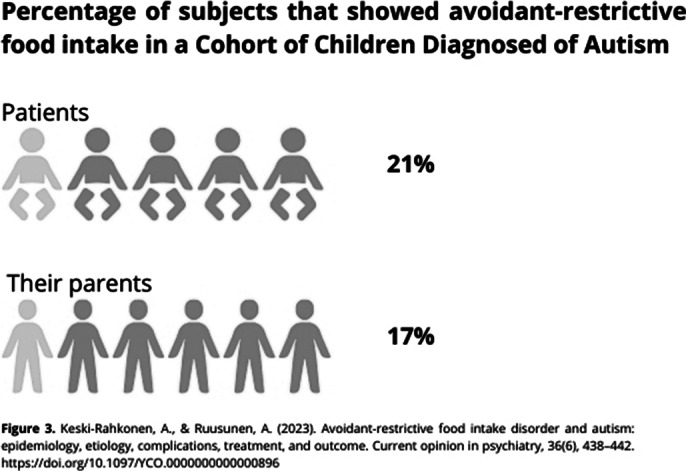

**Conclusions:**

The relationship between ASDs and EDs is common. Brede et al. (2020) proposed that certain autism traits, such as sensory sensitivities, social difficulties, identity issues, and the need for control, may contribute to restrictive eating behaviors. Westwood et al. (2018) [Figure 2] found a high prevalence of autism symptoms in adolescents with severe anorexia nervosa. Additionally, studies like Keski-Rahkonen et al. (2023) [Figure 3] report a significant prevalence of Avoidant/Restrictive Food Intake Disorder (ARFID) in individuals with ASD and their relatives. Further research is crucial to improve treatment approaches for these comorbid conditions.

**Disclosure of Interest:**

None Declared

